# Characterization of the relationship between neutron production and thermal load on a target material in an accelerator-based boron neutron capture therapy system employing a solid-state Li target

**DOI:** 10.1371/journal.pone.0225587

**Published:** 2019-11-22

**Authors:** Satoshi Nakamura, Hiroshi Igaki, Masashi Ito, Hiroyuki Okamoto, Shie Nishioka, Kotaro Iijima, Hiroki Nakayama, Mihiro Takemori, Shoji Imamichi, Tairo Kashihara, Kana Takahashi, Koji Inaba, Kae Okuma, Naoya Murakami, Yoshihisa Abe, Yuko Nakayama, Mitsuko Masutani, Teiji Nishio, Jun Itami

**Affiliations:** 1 Department of Medical Physics, National Cancer Center Hospital, Chuo-ku, Tokyo, Japan; 2 Division of Research and Development for Boron Neutron Capture Therapy, National Cancer Center Exploratory Oncology Research & Clinical Trial Center, Chuo-ku, Tokyo, Japan; 3 Department of Radiation Oncology, National Cancer Center Hospital, Chuo-ku, Tokyo, Japan; 4 Department of Radiology, National Center for Global Health and Medicine, Shinjuku-ku, Tokyo, Japan; 5 Department of Radiological Science, Graduate School of Human Health Sciences, Arakawa-ku, Tokyo, Japan; 6 Lab of Collaborative Research, Division of Cellular Signaling, National Cancer Center Research Institute, Chuo-ku, Tokyo, Japan; 7 Department of Radiological Technology, National Cancer Center Hospital, Chuo-ku, Tokyo, Japan; 8 Center for Bioinformatics and Molecular Medicine, Department of Frontier Life Sciences, Nagasaki University Graduate School of Biomedical Sciences, Sakamoto, Nagasaki, Japan; 9 Department of Medical Physics, Graduate School of Medicine, Tokyo Women’s Medical University, Shinjuku-ku, Tokyo, Japan; Los Alamos National Laboratory, UNITED STATES

## Abstract

An accelerator-based boron neutron capture therapy (BNCT) system that employs a solid-state Li target can achieve sufficient neutron flux derived from the ^7^Li(p,n) reaction. However, neutron production is complicated by the large thermal load expected on the target. The relationship between neutron production and thermal load was examined under various conditions. A target structure for neutron production consists of a Li target and a target basement. Four proton beam profiles were examined to vary the local thermal load on the target structure while maintaining a constant total thermal load. The efficiency of neutron production was evaluated with respect to the total number of protons delivered to the target structure. The target structure was also evaluated by observing its surface after certain numbers of protons were delivered. The yield of the sputtering effect was calculated via a Monte Carlo simulation to investigate whether it caused complications in neutron production. The efficiency of neutron production and the amount of damage done depended on the proton profile. A more focused proton profile resulted in greater damage. The efficiency decreased as the total number of protons delivered to the target structure increased, and the rate of decrease depended on the proton profile. The sputtering effect was not sufficiently large to be a main factor in the reduction in neutron production. The proton beam profile on the target structure was found to be important to the stable operation of the system with a solid-state Li target. The main factor in the rate of reduction in neutron production was found to be the local thermal load induced by proton irradiation of the target.

## Introduction

Based on the results of in vivo and in vitro experiments, boron neutron capture therapy (BNCT) is expected to kill cancer cells that are resistant to conventional radiotherapies, such as photon therapy [1–5]. This is because compounds containing ^10^B are delivered to the cancer cells, which are then killed by the ^10^B(n, α)^7^Li reaction caused by neutron irradiation [2, 6]. Based on the results of such studies, clinical trials of BNCT have been conducted and their outcomes reported [7–9]. Most such trials have been performed using research nuclear reactors such as that at Kyoto University [7–9]. However, nuclear-reactor-based BNCT is difficult to implement as a standard treatment method because it is difficult to install a research reactor in a hospital. Hence, a neutron source that can substitute for a research reactor is needed to put BNCT into practice.

According to recent studies [10–15], accelerator-based BNCT systems can achieve sufficient neutron flux to treat patients and can be installed in hospitals. In most such systems, neutrons are generated by the reaction between accelerated protons and a target material. The generated neutrons are moderated to a suitable energy spectrum for BNCT after passing through beam-shaping assemblies [16–17]. Depending on the neutron generation method, the systems that have been constructed for BNCT can be divided into two types. In each system, the energy of the accelerated protons and the target material are different. One type of system employs solid-state beryllium as the target material, and the protons have an energy greater than 8 MeV [11–12]. BNCT has been conducted using this type of system [12]. The other type of system employs solid-state lithium as the target material, and the proton energy is in the range of 2.0–3.0 MeV [10, 13–15]. An advantage of the latter type of system is the lower energy of the generated neutrons, which facilitates implementation in a relatively compact BNCT system, because a suitable neutron energy spectrum for BNCT can be acquired without a thick moderator. However, a disadvantage of the latter type of system is the melting point of the target material, lithium, which is lower than that of beryllium. Accelerator-based BNCT systems require a high thermal loading of the target material to achieve sufficient neutron flux for BNCT [10]. The latter type of system has been installed at the National Cancer Center Hospital (NCCH) in Tokyo, Japan.

Previous studies have suggested that in systems that employ solid-state Li targets, degradation of the target material under proton bombardment should be expected due to ion impact, the high operating temperature of the target material, and other factors. This degradation may reduce the efficiency of neutron production [18–19]. Hence, it is important that the efficiency of neutron production is evaluated under various thermal loading conditions (i.e., proton profiles). This study was conducted to examine the relationship between the efficiency of neutron production and the thermal load on the target material in an accelerator-based BNCT system employing a solid-state Li target.

## Materials and methods

**Fig 1:**
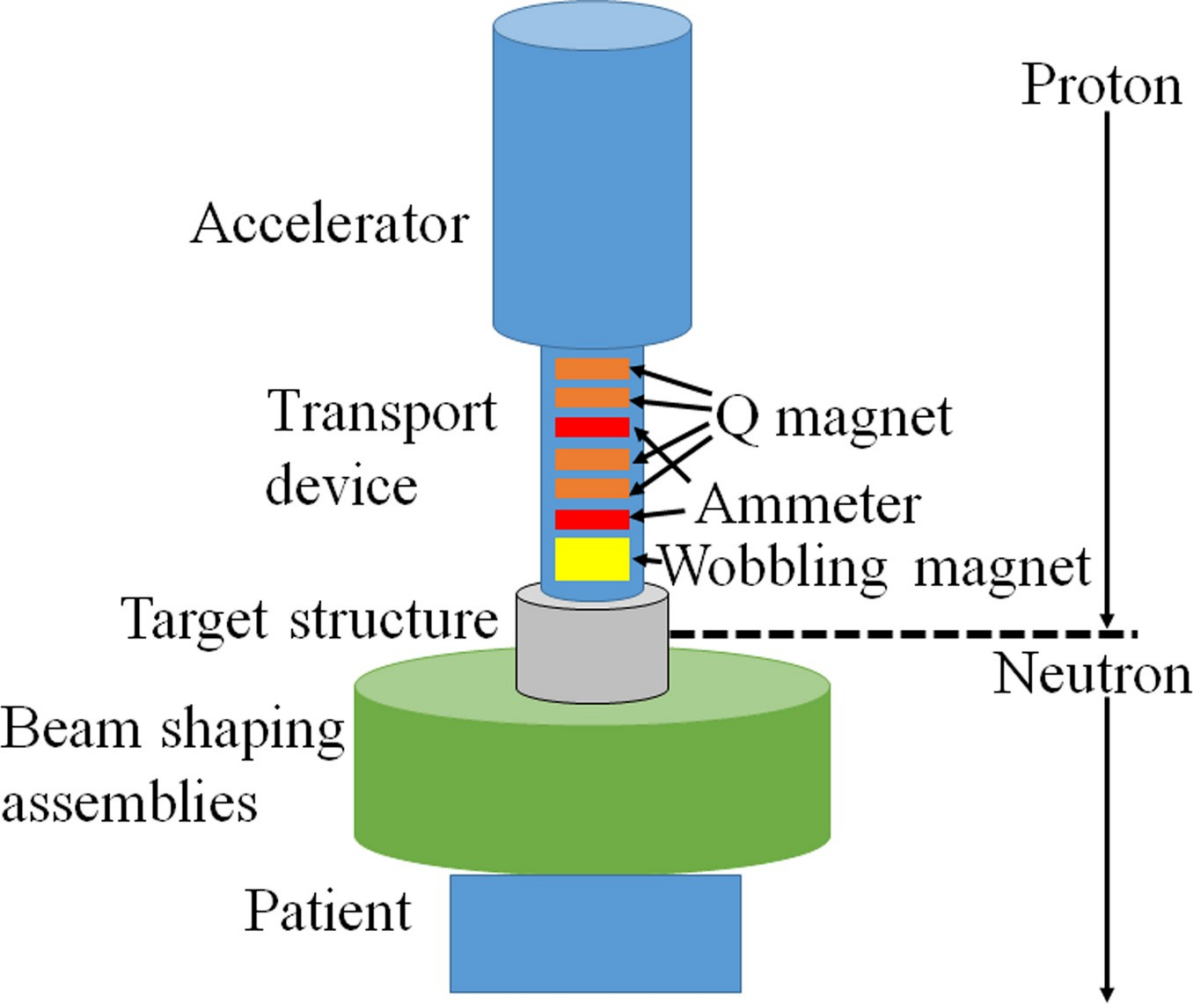
Schematic illustration of the accelerator-based BNCT system.

**Fig 2:**
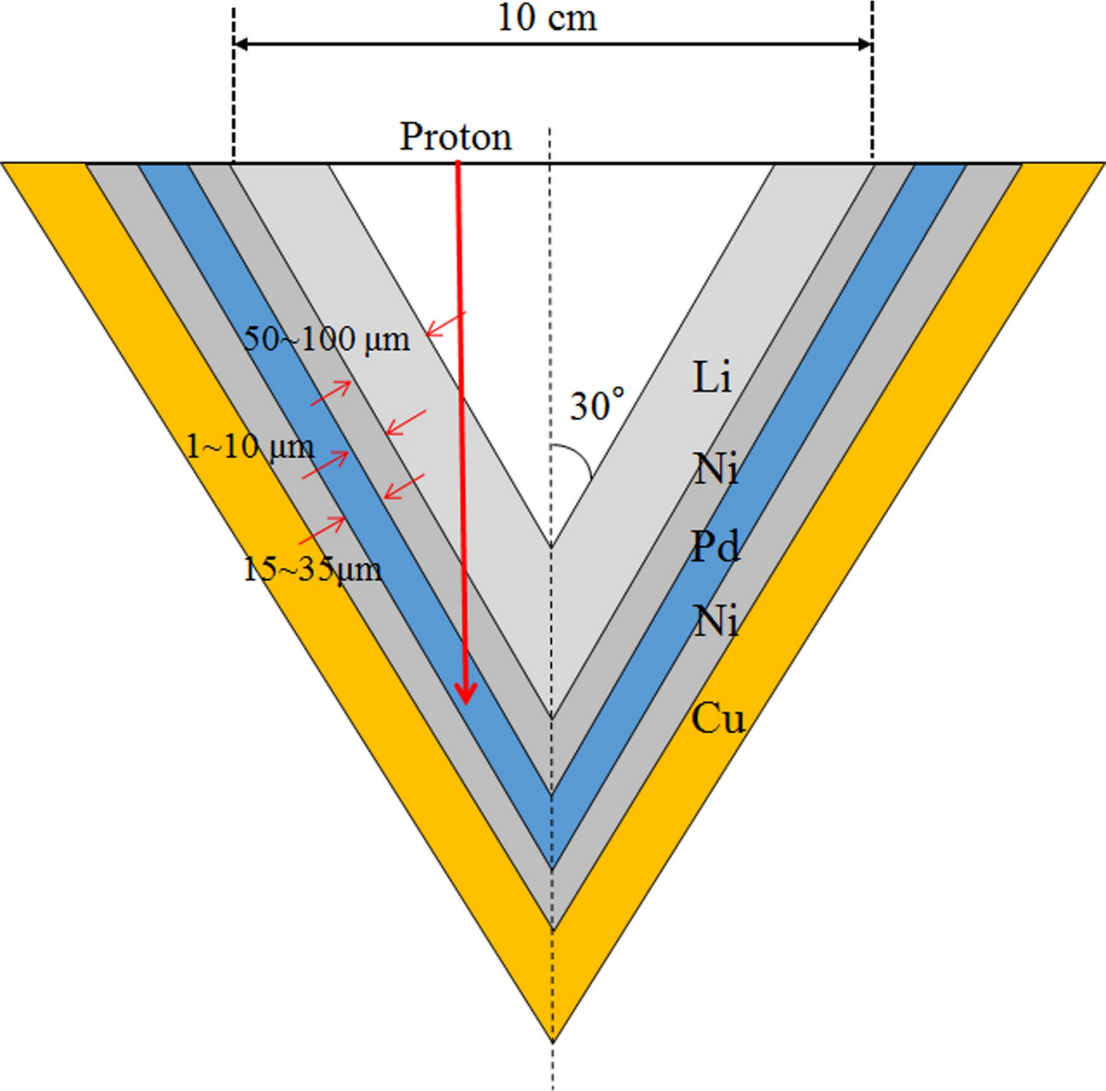
Schematic illustration of the target structure.

Experiments were performed in the accelerator-based BNCT system (manufactured by Cancer Intelligence Care Systems, Inc., Tokyo, Japan) at NCCH. The system consists of a proton accelerator, transport devices for the accelerated protons, a target structure (containing the Li target), and a beam shaping assembly (BSA). A schema of the system is shown in Fig 1. Accelerated protons with a nominal mean energy of 2.5 MeV and a maximum proton current of 20 mA are delivered to the target structure to achieve sufficient neutron flux. After passing through the BSA, the generated neutrons are moderated to a suitable energy level for BNCT. The target structure consists of a thin solid-state Li target, a first Ni layer, a Pd layer, a second Ni layer, and a copper support. The thicknesses of the Li target, first Ni layer, Pd layer, second Ni layer, and copper support are 50.0, 3.0, 14.0, 7.0, and 10.0 × 10^2^ μm, respectively. A schema of the target structure is shown in Fig 2. The Pd layer is incorporated into the design of the target structure to avoid blistering, which would induce modification of the physical properties of the Li target (i.e., the surface of the target structure). Most of the protons are absorbed by the Pd layer because Pd has a very high hydrogen diffusivity [20]. According to previous studies, degradation of the Li target is induced by high operating temperatures, and the efficiency of neutron generation is reduced as a result of thinning of the Li target [19]. Thus, in this study, the efficiency of neutron generation was evaluated with respect to the total number of protons delivered to the Li target.

The cooling efficiency of the target structure is important because the maximum thermal load on the target structure can reach 50 kW. Hence, a cooling system is employed to reduce the thermal load on the target structure. There are three main strategies for reducing the thermal load. One strategy is to set the incident angle of the delivered protons with respect to the target structure to 30° (Fig 2). This improves the efficiency of cooling the target structure because the local thermal load on the target structure decreases as the incident area of the delivered protons on the target structure increases. Another strategy is to use a water cooling apparatus for the target structure. One such apparatus passes cooling water to the target structure through the copper support, at a rate of 250 L/min. The last strategy involves proton irradiation. A wobbling technique is used to deliver protons to the target structure. The proton beam is enlarged with wobbling magnets, which are equipped with transport devices (Fig 1). Hence, an enlarged proton beam can be delivered to the target structure. The frequency of the wobbling is 37 Hz.

Given the possibility of fatigue of the target structure due to the high thermal loads it receives, the target structure should be replaced with another after a certain number of protons (less than 800 mA×h) have been delivered to it. Additionally, in an accelerator-based BNCT system employing a Li target, ^7^Be is induced by the reaction of ^7^Li(p,n)^7^Be, and its nuclide emits a gamma ray with an energy of 478 keV per decay. It is crucial to prevent staff from unexpected exposure as a result of the induced radionuclide when replacing the target structure. Hence, the system is designed to wash away both the Li target and the induced radionuclide (^7^Be) after a certain number of protons are delivered to the target structure.

To evaluate the relationship between the efficiency of neutron production and the pattern of the thermal load to the target structure, four proton profiles were examined in this study, and the target structure was replaced after each examination. The proton profile was adjusted to produce the intended profile by changing the intensity of four Q magnets on the transport device (Fig 1). The efficiency of neutron production and the condition of the target structure after bombardment by the protons were evaluated for each target structure.

### Proton profile measurement

**Fig 3:**
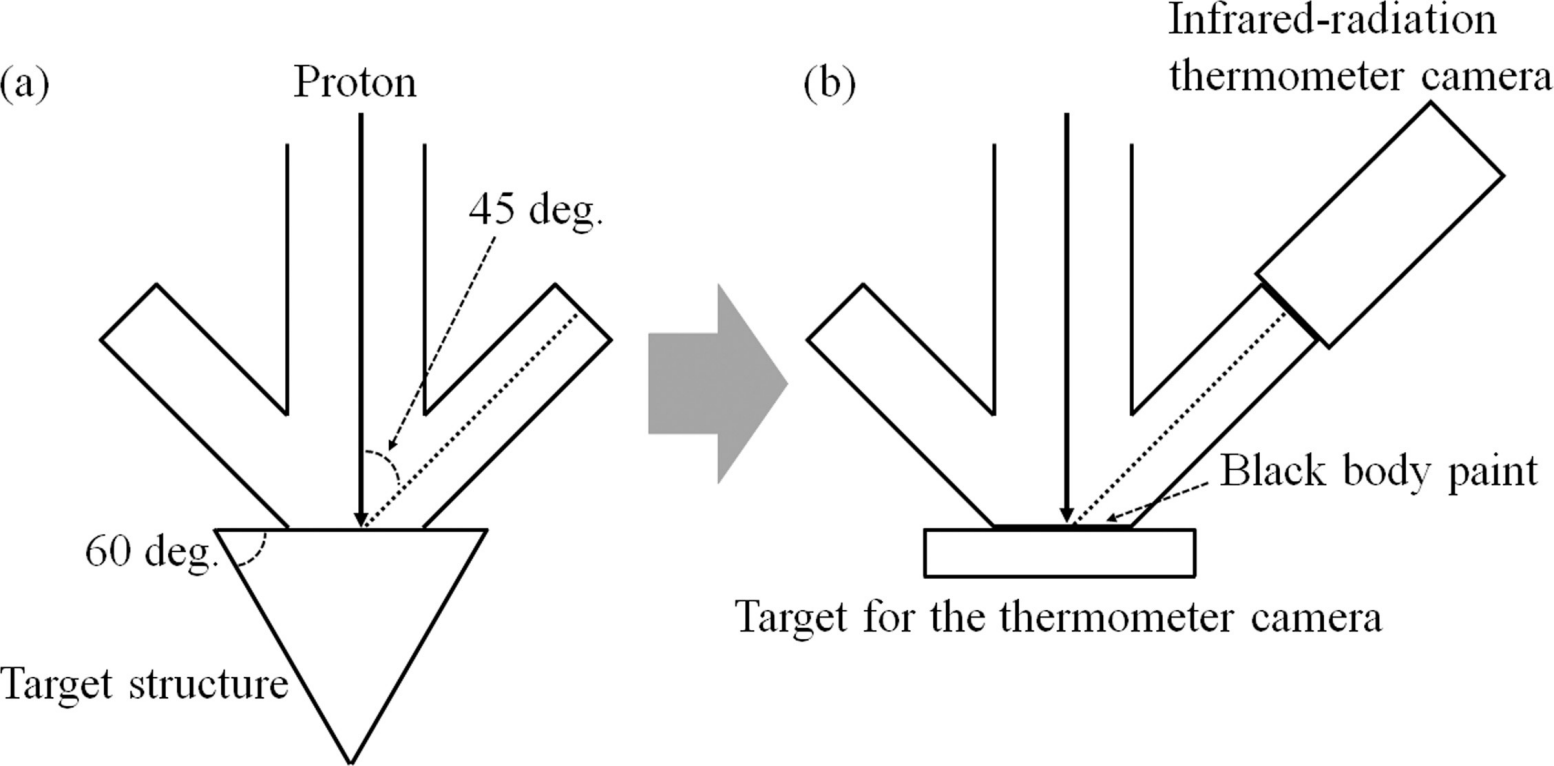
Setup around the target in the accelerator-based BNCT system. (a) The setup of the target structure for the accelerator-based BNCT system and (b) the setup of the target for the thermometer camera.

**Fig 4:**
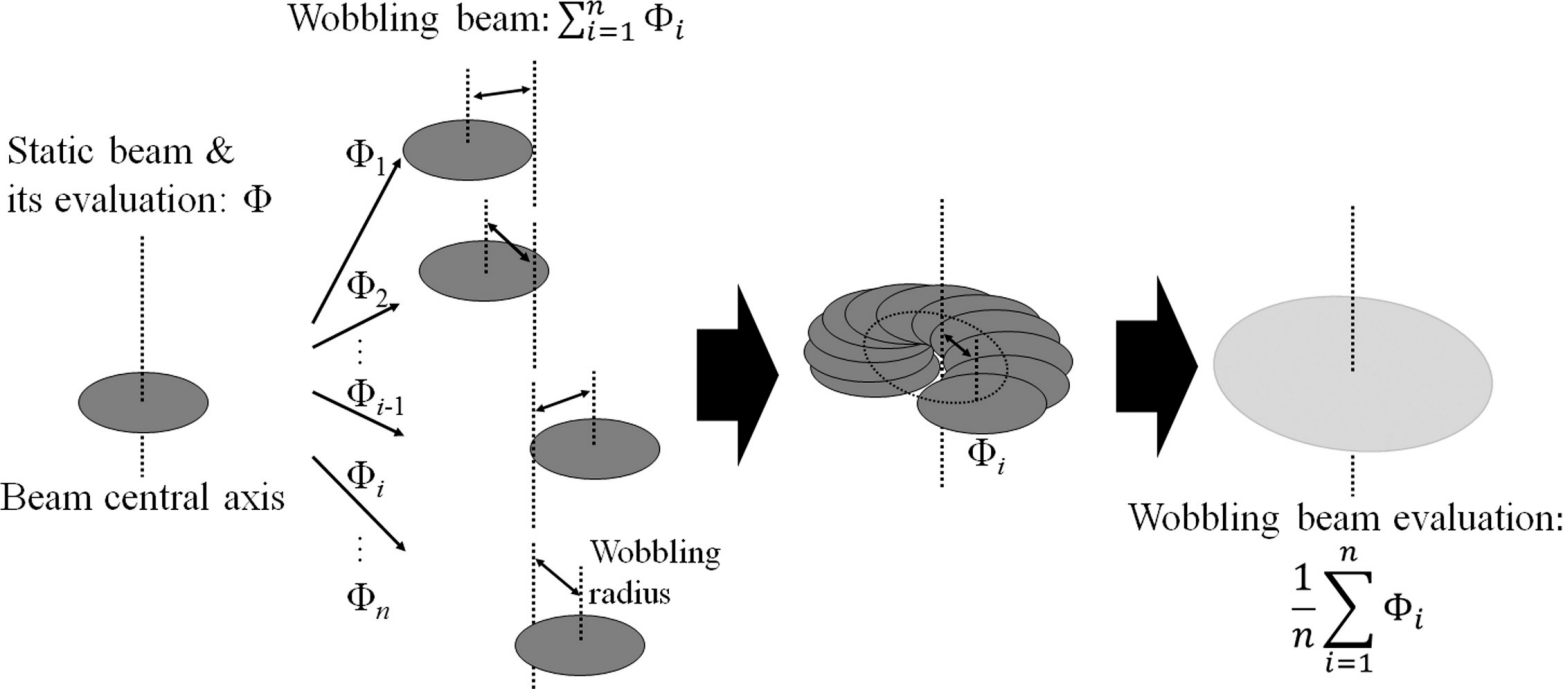
Schematic illustration of evaluation of the proton profile. (a) The static beam evaluation and (b) the wobbling beam evaluation.

The proton profile was measured with an infrared radiation thermometer camera (G100, Nippon Avionics Co., Ltd.). To measure the proton profile, the target structure in the BNCT system was replaced with a target for the thermometer camera. This target consisted of a copper target with a painted black body and a cooling apparatus. The copper target was placed perpendicular to the proton beam. Its cooling apparatus provided flowing water to the copper target at a rate of 15 L/min. Proton profiles were measured on the copper target by observing the copper target temperature using the thermometer camera [21]. Heat creates thermal excitation of the copper target, which then emits infrared radiation. This infrared radiation was detected by the thermometer camera, and the proton profile on the copper target was then evaluated using the temperature distribution. The setup for the experiment is shown in Fig 3. All proton profile measurements were performed using this setup. Because the viewing angle of the thermometer camera with respect to the proton beam was set to 45°, the measured profile was compressed laterally by $1/\sqrt{2}$. Thus, the angle effect was eliminated in the evaluation of the proton profile. The proton beam was evaluated with and without wobbling (i.e., with a wobbling beam and a static beam), even though only a wobbling beam is used in the standard operation of an accelerator-based BNCT system with a Li target. The reason for this is that a wobbling beam is produced by a static beam under the action of sequential wobbling. Therefore, both static and wobbling beams were evaluated to examine whether each of them affected the accelerator-based BNCT system. To evaluate the proton profile of a wobbling beam, the temperature in each area of the copper target was during each wobbling sequence was determined, and the average temperature was calculated. A schema of the evaluation of the wobbling beam is shown in Fig 4. The frame rate of the thermometer camera was 30 Hz, which was not equal to the frequency of wobbling.

### Measurement of neutron production efficiency

**Fig 5:**
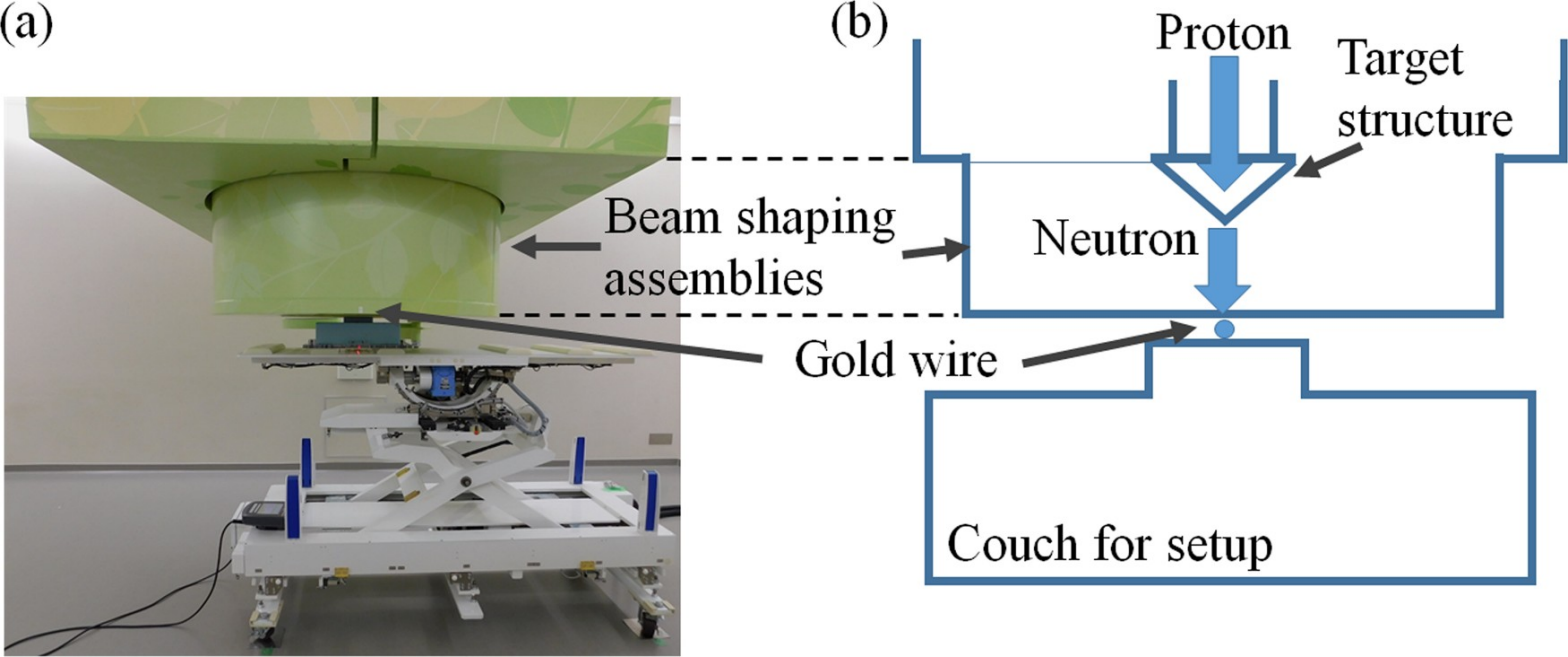
Measurement geometry for neutron irradiation on gold. (a) A picture of the actual measurement setup and (b) measurement schematic.

According to previous research, the efficiency of neutron production is decreased by the delivery of protons to the target structure as a result of thinning of the Li target that occurs because of the thermal load on the target structure [18–19]. Thus, the efficiency of neutron production was evaluated in this study with respect to the total number of protons delivered to the target structure. In each evaluation interval, the accumulated proton charges delivered to the target structure did not exceed 86.4 × 10^3^ mC, which is comparable to 2 h of irradiation with a proton current of 12.0 mA. It is well known that saturated radioactivity is proportional to neutron flux. Hence, the neutron production efficiency was evaluated in this study by measuring the saturated radioactivity of a gold wire with a 0.5-mm diameter and 10.0-mm length. The purity of the gold wire was 99.5%. Fig 5 shows the measurement geometry. The gold wire was placed at the center of the bottom surface of an irradiation port of the accelerator-based BNCT system. In each measurement, the number of protons delivered to the target structure was approximately 3.6 × 10^3^ mC. After neutron exposure, a gold wire induces ^198^Au, which emits a 412-keV gamma ray. In this study, the number of emitted gamma rays was counted using a high-purity germanium (HP-Ge) detector, and the induced radioactivity of ^198^Au within the gold wire was determined. The detection efficiency of the HP-Ge detector for the induced ^198^Au within the gold wire was calculated via Monte Carlo simulation (GEANT4, ver. 10.1 [22]). The measurement geometry for the induced radioactivity was reproduced in the simulation to calculate the detection efficiency. The HP-Ge detector has been modeled using radioactive sources validated by the Japan Radioisotope Association of Tokyo, Japan and has been reported in a previous study [23]. The events resulting in the reactions between the induced radioactivity and the HP-Ge detector were counted using a multichannel analyzer (MCA7600, Seiko EG&G, Tokyo, Japan). Gamma Studio (Seiko EG&G, Tokyo, Japan) was used to count the number of photoelectric events that occurred in the HP-Ge detector.

In an accelerator-based BNCT system, neutrons are produced by the reaction between accelerated protons and the target material. Hence, the number of generated neutrons depends on the number of protons delivered to the target structure. To investigate the relationship between the number of neutrons and the number of protons delivered to the target structure, equations proposed in a previous study were used to evaluate the saturated radioactivity [19]. During the neutron exposure of the gold wire, the number of protons delivered to the target structure was measured with an ammeter (NPCT-CF6, Bergoz Instrumentation, Saint-Genis-Pouilly, France) equipped with a transport device. The saturated radioactivity of ^198^Au within the gold wire was evaluated in units of Bq/mA to eliminate the effect of fluctuation in the number of protons delivered.

### Target observation

To evaluate the effects of proton delivery to the target structure, the surface of the target structure to which a certain number of protons was delivered was observed before and after the Li target was washed away.

### Evaluation of the sputtering effect between the delivered protons and the Li target

It is well known that sputtering occurs when charged particles are delivered to a solid-state target [24–25]. In this study, the sputtering yield on the Li target was calculated via Monte Carlo simulation (TRIM, SRIM 2013-pro). The target structure was reproduced in the simulation. To calculate the sputtering yield, protons with energy levels of 2.40, 2.45, and 2.50 MeV were delivered to the reproduced target structure.

## Results

### Proton profile measurement

**Fig 6:**
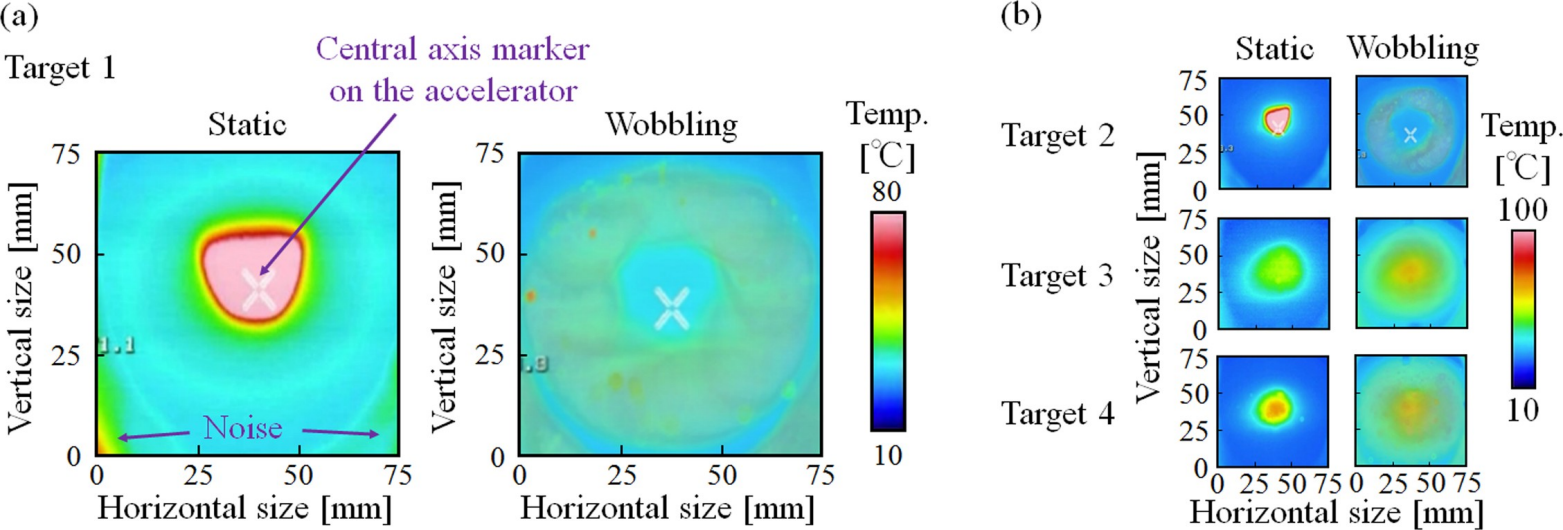
Measurement results for each proton profile obtained using the thermometer camera. (a) Proton profile for target structure 1 and (b) target structures 2, 3, and 4. Although these results are only for wobbling beams applied to the target structure, static beams were also evaluated in this study.

Four static and wobbling beam patterns were selected after tuning of the proton profiles. Hence, the proton profile applied was different for each target structure. The static and wobbling beams are shown in Fig 6. The parameters of the static and wobbling beams are summarized in Tables 1 and 2, respectively. A focused beam was applied to target structures 1 and 2, whereas a defocused beam was applied to target structures 3 and 4. For the wobbling-beam profiles, there was a non-proton-delivered area around the centers of target structures 1 and 2 that did not exist for target structures 3 and 4. When the static beam profile was not circular in shape, the wobbling beam profile was not circular in shape (Fig 6). As a result, the wobbling beam had a local high-temperature region in its profile.

**Table 1:** Parameters of the static beam profile for each target.

Target No.	Static beam diameter (Horizontal direction)	Static beam diameter (Vertical direction)	Maximum temperature
1	38.0 mm	39.0 mm	> 80°C *1
2	20.0 mm	21.0 mm	> 100°C *1
3	40.0 mm	38.5 mm	71°C
4	56.5 mm	60.0 mm	82°C

*1: Out of measurement range

**Table 2:** Parameters of the wobbling beam profile for each target.

Target No.	Wobbling beam diameter (Horizontal direction)	Wobbling beam diameter (Vertical direction)
1	64.9 mm	68.0 mm
2	58.0 mm	56.0 mm
3	74.0 mm	71.0 mm
4	75.0. mm	75.0 mm

### Measurement of neutron production efficiency

**Fig 7:**
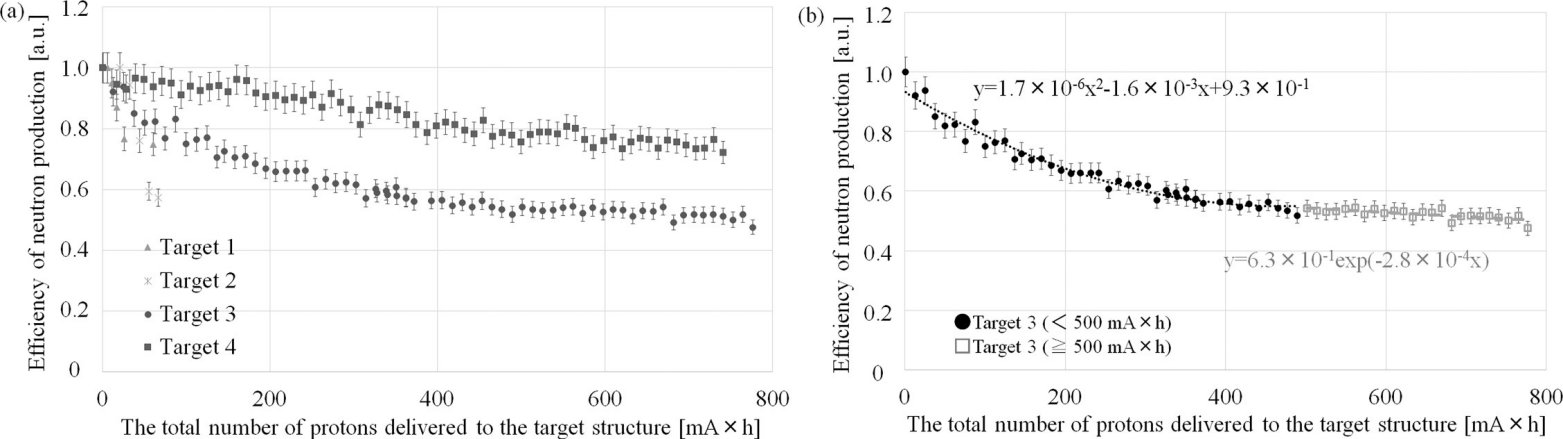
Relationship between efficiency of neutron production and the number of protons delivered to each target structure. (a) Relationship for target structures 1–4 and (b) focus on relationship for target structure 3.

The efficiency of neutron production was measured for each target structure. For all of the target structures, the efficiency of neutron production decreased as the total number of protons delivered to the target structure increased. However, the rate of reduction was different for each target structure. The efficiency with respect to the total number of protons delivered to each of the target structures is shown in Fig 7. For target structures 1 and 2, the efficiency decreased drastically during an early period in the proton irradiation of the target structure. For target structure 3, two rates for the reduction of the neutron production efficiency were observed: an initial rate that could be expressed as a quadratic function (~500 mA×h) and a subsequent rate that could be expressed an exponential function (beyond 500 mA×h). For target structure 4, the rate of reduction did not depend on the total number of protons delivered to the target structure.

### Target observation

**Fig 8:**
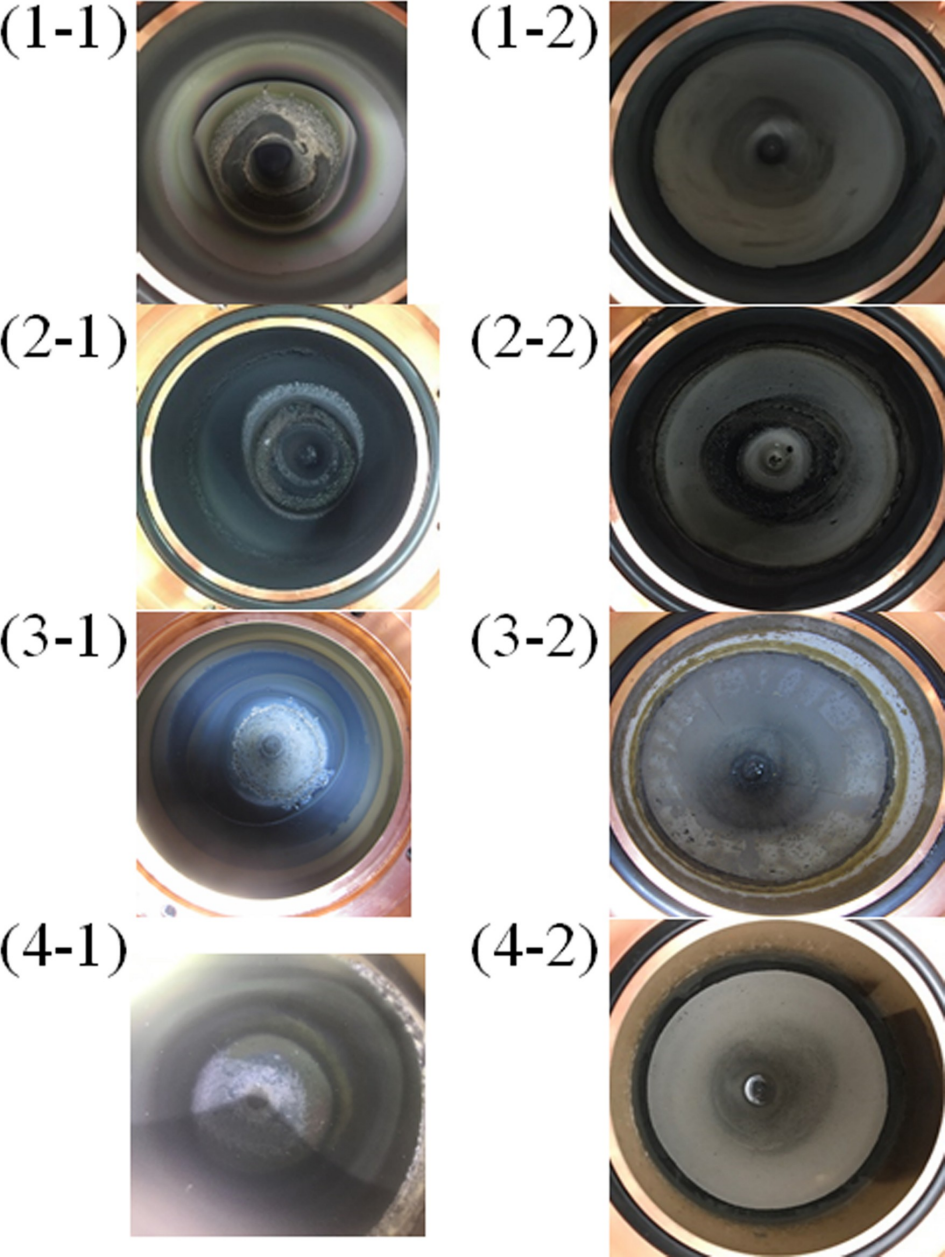
Pictures of the surface of each target structure before and after washing away of the Li target. A certain number of protons were delivered to each target structure. In the caption notation “(*x*-*y*)”, “x” is the target structure number and “y” indicates before (“1”) or after (“2”) washing away of the Li target. The apparent angle of the target structure in Fig 8 corresponds to that in Fig 6. Target structure 4 wrapped in plastic before the Li target was washed away to avoid contact with the radioactive contaminant. Hence, the (4–1) results are not clear.

The surface conditions of the target structures after delivery of certain numbers of protons before and after washing away of the Li target are shown in Fig 8. The apparent angle of the target structure shown in Fig 8 corresponds to that in Fig 6. It should be noted that target structure 4 was wrapped in plastic to avoid contact with the radioactive contaminant induced by the radionuclide of ^7^Be. The surface conditions were different for each target structure. The target conditions and proton profile for each target structure are summarized in Table 3. All the target structures had a disturbance region on the Li target before washing away of the Li target. Two of the target structures (1 and 2) exhibited remarkable damage after washing away of the Li target; the other two (3 and 4) did not exhibit much damage.

**Table 3:** Target condition in each of four targets.

Target structure	Total number of delivered protons [mA×h]	Target condition at the end of irradiation*1
1	148.08	Damage
2	74.93	Break
3	1598.9	No damage
4	1078.3	No damage

*1: The target structures were examined visually after washing away of the Li layer. “Break” means no capability for irradiation because of degradation of the degree of vacuum. “Damage” means the state that can be irradiated but with roughness observed on the surface of the target support foundation. “No damage” means the state that can be irradiated without surface roughness.

### Evaluation of the sputtering effect between the delivered protons and the Li target

The sputtering yield in the accelerator-based BNCT system with a solid-state Li target was evaluated by means of simulation. The sputtering yields for incident proton energies of 2.40, 2.45, and 2.50 MeV were 4.0 ± 0.4 × 10^−6^, 3.3 ± 0.4 × 10^−6^, and 2.9 ± 0.4 × 10^−6^ atoms/ion, respectively.

## Discussion

This study focused on the relationship between the efficiency of neutron production and the proton profile applied to the target structure in an accelerator-based BNCT system with a solid-state Li target. Ultimately, the goal of this investigation of key parameters related to the operation of the system and its neutron production was to gain insights into how to make the best clinical use of this type of system. The results of this study show that the proton profile on the target structure is a critical parameter in the stable operation of the system in terms of neutron production. This is because a large local thermal load on the target structure is required to achieve sufficient neutron flux in the system. A focused beam profile induces a higher local thermal load on the target structure than a defocused beam profile, even when the total thermal load on the target structure (i.e., the number of protons delivered) is the same. According to Figs 6 and 8, A high temperature produced by a wobbling beam corresponds to a larger disturbance region on the surface of the Li target before the target is washed away. Additionally, after the Li target was washed away, remarkable damage was observed in cases in which focused proton beams were applied to the target structures (1 and 2). The locations of damage on target structures 1 and 2 also corresponded to a high temperature region in the wobbling beam profile (Figs 6 and 8). On the other hand, according to Figs 6 and 8, when defocused proton beams were applied to target structures (3 and 4), the damage observed was not remarkable. Additionally, according to Fig 7, the efficiency of neutron production was reduced as the total number of protons delivered to the target structure increased. The observed efficiency reduction is consistent with that observed in a previous study [19].

Differences in the rate of neutron production efficiency among the target structures were also observed in this study. Defocused proton beams (i.e., lower proton concentrations) were applied to target structures 3 and 4, whereas focused proton beams were applied to target structures 1 and 2. The rates of reduction for structures 3 and 4 were lower than those for target structures 1 and 2. These results suggest that the degree of reduction of the efficiency of neutron production and the degree of damage to the target structure after proton irradiation depend on the local thermal load to the target structure rather than the total thermal load. In this study, although the total thermal load to each of the target structures was the same, differences were observed among the target structures in the degree of damage and the reduction in the rate of efficiency of neutron production. The degree of damage and the reduction in the efficiency rate were both found to be associated with the degree of concentration of the proton delivery to the target structure. A uniform thermal load to the target structure is typically considered when a thermal load is discussed [10]. However, it is difficult to produce a proton beam that delivers a uniform number of protons to each portion of the delivery area. Hence, high local thermal loads on the target structure must be considered in addition to the total thermal load. Thus, control of the proton profile is important not only for wobbling beams but also for static beams in accelerator-based BNCT systems. In addition, local high temperature regions and non-circular beam shapes were observed with the wobbling beam when the static beam profile was not tuned adequately (Fig 6), and a remarkable degree of damage to the target structure was then observed (Fig 8). The regions of the target structures exhibiting the greatest damage corresponded to those exhibiting the highest temperatures. Therefore, careful control of the thermal load on the target structure is crucial to the performance of an accelerator-based BNCT system.

Because, as Nakamura *et al*., Kobayashi *et al*., and Lee *et al*. have suggested, the efficiency of neutron production depends on the thickness of the Li target [15, 18–19, 26], a reduction in the efficiency of neutron production can be induced by thinning of the Li target. According to Fig 7, three patterns in the rate of reduction were observed in this study, one of which was a drastically decreased rate of reduction during an early period in the delivery of protons to the target structures (1 and 2). The other two patterns (observed with structures 3 and 4) were a rate of reduction in the efficiency of neutron production that could be expressed as a quadratic function (during an early period for target structure 3, < 500 mA×h) and a rate that could be expressed as an exponential function (for target structure 4 and during a later period for target structure 3, ≥ 500 mA×h). The diameters of both the static and wobbling beams for target structure 4 were larger than those for target structure 3. Hence, the cooling efficiency was better for target structure 4 and the rate of reduction of neutron production efficiency was lower for target structure 4. For target structure 3, the thickness of the Li target may have decreased considerably until the temperature rise and cooling efficiency of the target structure reached a certain equilibrium. After a certain number of protons were delivered to the target structure, the efficiency of neutron production was reduced exponentially in target structure 3 (above 500 mA×h). In target structure 4, the thinning of the Li target throughout the evaluation period might have been due to the efficiency of neutron production being reduced at a constant rate after equilibrium. Hence, the rate of efficiency reduction with respect to the total number of protons delivered to the target structure may be different for different accelerator-based BNCT systems because the cooling efficiency depends on the characteristics of each individual system. The results of this study suggest that observing the reduction in neutron production efficiency is important to not only maximizing the efficiency of neutron production but also optimizing the characteristics of the target structure.

Previous studies have suggested that charged particle irradiation focused on a solid-state target induces sputtering [24–25] and that this might induce a reduction in the efficiency of neutron production. In this study, sputtering effects were examined via simulation. Sputtering effects may be associated with a reduction in the efficiency of neutron production. However, sputtering was not found in this study to have a notable effect on the efficiency of neutron production. As the results for target structure 4 in Fig 7 show, the reduction in the efficiency of neutron production was approximately 30% when the total number of protons delivered to the target structure reached 700 mA×h. In a previous study [19], the thickness of an Li target was decreased by approximately 20–30 μm during proton irradiation of 700 mA×h. Because proton irradiation of 700 mA×h is equal to 2.52×10^6^ mC, the yield of sputtering is approximately 4.6 ± 0.6 × 10^15^ atoms. Thus, weight of the sputtered Li is approximately 5.3 × 10^−7^ g. If the proton irradiation area on an Li target is assumed to be 2.1 × 10^2^ cm^2^ (using the proton profile for target structure 4), the thickness of the Li target is decreased by approximately 4.6×10^−5^ μm. Therefore, the main reason for the reduction in the efficiency of neutron production is the thermal load applied to the target structure rather than the sputtering effect.

According to previous reports [27–29], events related to thermal loading of a target structure occur during the production of a radiolabelled compound used in positron emission tomography (PET). To produce the compound, accelerated protons are delivered to a target structure. In these previous studies, the yield of this compound suffered from heating of the target structure. It has been reported that the yield of the compound is increased by improving the cooling efficiency of the target structure [28–29]. Hence, an accelerator-based BNCT system that employs a solid-state Li target may not be able to avoid a reduction in the efficiency of neutron production, although the degree of reduction depends on the ability to eliminate the thermal load on the target structure. Thus, it is important that the reduction in efficiency of neutron production be considered for each such system. The effect of the thickness of the Li target needs to be considered because the number of neutrons generated depends on both its thickness and the incident proton energy. One of the latter factors was examined in a previous study [19]. When the incident proton energy was less than or equal to 2.5 MeV, no remarkable effect, in terms of BNCT doses, was observed. However, because the reduction of efficiency during the treatment was not discussed, which is important for the system, a discussion about the same will be conducted in the future study.

## Conclusions

An accelerator-based BNCT system that employs a solid-state Li target has been developed for installation in a hospital. In this system, both the thermal load on the target structure and sputtering of the Li target due to proton irradiation may reduce the efficiency of neutron production, although the main reason for the reduction in efficiency is the thermal load on the target structure. The thermal load may also induce a remarkable degree of damage in the target structure and a reduction in the efficiency of neutron production. However, tuning the proton beam profile adequately can prevent damage to the target structure and minimize reduction of the efficiency of neutron production. The results of this study also indicate that applying a larger proton profile in an accelerator-based BNCT system helps to achieve stable operation of the system and minimize reduction of the neutron output. Both the instantaneous thermal load (i.e., the static beam) applied to the target structure and the averaged thermal load (i.e., the wobbling beam) are important to the performance of the system. Therefore, if the local thermal load on the target structure is adequately considered, damage to the target structure can be prevented and stable operation of an accelerator-based BNCT system with a solid-state Li target can be expected. Although the cooling efficiency was only considered in case of the accelerator-based BNCT system in NCCH, the thermal loads are important to compensate the disadvantage of a Li target in an accelerator-based BNCT system employing a solid-state Li target. Additionally, for an accelerator-based BNCT system employing a solid-state Li target, it is necessary to discuss the reduction of efficiency during the treatment considering the thermal loads on the target structure. This will be a part of future study.
